# Effect of saliva fluid properties on pathogen transmissibility

**DOI:** 10.1038/s41598-021-95559-6

**Published:** 2021-08-06

**Authors:** Jonathan Reyes, Douglas Fontes, Alexander Bazzi, Michelle Otero, Kareem Ahmed, Michael Kinzel

**Affiliations:** 1 Mechanical and Aerospace Engineering, University of Central Florida, Orlando, FL 32766 USA; 2 Florida Space Institute, University of Central Florida, Orlando, FL 32766 USA

**Keywords:** Viral infection, Optical imaging

## Abstract

With an increasing body of evidence that SARS-CoV-2 is an airborne pathogen, droplet character formed during speech, coughs, and sneezes are important. Larger droplets tend to fall faster and are less prone to drive the airborne transmission pathway. Alternatively, small droplets (aerosols) can remain suspended for long time periods. The small size of SARS-CoV-2 enables it to be encapsulated in these aerosols, thereby increasing the pathogen’s ability to be transmitted via airborne paths. Droplet formation during human respiratory events relates to airspeed (speech, cough, sneeze), fluid properties of the saliva/mucus, and the fluid content itself. In this work, we study the fluidic drivers (fluid properties and content) and their influence on factors relating to transmissibility. We explore the relationship between saliva fluid properties and droplet airborne transmission paths. Interestingly, the natural human response appears to potentially work with these drivers to mitigate pathogen transmission. In this work, the saliva is varied using two approaches: (1) modifying the saliva with colloids that increase the viscosity/surface tension, and (2) stimulating the saliva content to increased/decreased levels. Through modern experimental and numerical flow diagnostic methods, the character, content, and exposure to droplets and aerosols are all evaluated. The results indicate that altering the saliva properties can significantly impact the droplet size distribution, the formation of aerosols, the trajectory of the bulk of the droplet plume, and the exposure (or transmissibility) to droplets. High-fidelity numerical methods used and verify that increased droplet size character enhances droplet fallout. In the context of natural saliva response, we find previous studies indicating natural human responses of increased saliva viscosity from stress and reduced saliva content from either stress or illness. These responses both favorably correspond to reduced transmissibility. Such a finding also relates to potential control methods, hence, we compared results to a surgical mask. In general, we find that saliva alteration can produce fewer and larger droplets with less content and aerosols. Such results indicate a novel approach to alter SARS-CoV-2’s transmission path and may act as a way to control the COVID-19 pandemic, as well as influenza and the common cold.

## Introduction

The 2020 COVID-19 pandemic is driven by an airborne transmitted pathogen, SARS-CoV-2^1–3^. The pathogen has led to large-scale infections, death, health-system overloads, and severe economic damage^4^. These circumstances demand to explore multidisciplinary mechanisms associated with SARS-CoV-2 transmission. Airborne transmission paths are associated with droplets formed from natural human respiratory functions such as sneezing, coughing, speaking, and breathing^5^.

The World Health Organization and other agencies, such as the Centers for Disease Control and Prevention, recommend two common techniques to reduce droplet-related pathogen transmission: social distancing and wearing face masks^6^. In essence, both aim to reduce droplets transmitted during human respiratory function. Previous experience implies that transmission of influenza virus tends to be associated with smaller droplets (aerosols)^7^ and that there is an underlying threshold in terms of quantity of viral transmission events^8^ associated with transmission. Hence, prevention of airborne pathogens demands reducing the exposure to pathogen-laden droplets and aerosols.

These pathogen-laden droplets are ejected from the host and, depending on the droplet size, can take various paths for transmission. A large droplet is heavy compared to its aerodynamic drag; hence, it essentially falls similar to the famous experiments of Galileo at the Leaning Tower of Pisa. Small droplets (or aerosols), are light relative to their aerodynamic drag. For these droplets, Galileo’s assumptions are violated; hence, the droplet can remain suspended, and even slight winds can lead the droplet to appear to defy gravity. According to Wells^5^, the time spent in the airborne transmission correlates to the time the droplet spends in the atmosphere involving a competition between settling time and the time required to evaporate. Larger droplets have short settling times ($<3$ s) and tend to fall to the ground. As droplets get smaller, at some point they evaporate prior to hitting the ground and become suspended aerosols^9^. In this state, influenza^7,8^ and SARS-CoV-2^10^ have been measured to survive as suspended for time periods as long as 2–3 h. Such droplet processes, potentially laden with pathogens, are one component of the pathogen transmission pathway.

In the context of sneezes, which is the focus of this work, the flow from a sneeze has also been well evaluated using advanced flow diagnostics. Some of the original work was developed using Schilieren imagery, which indicated the efficacy of masks^11^, as well as aerosol formation^12^. Additionally, studies also evaluated the influence of turbulence and thermal plumes^13,14^. These studies, along with an improved understanding of human respiration^15,16^, drive improved understanding of some of the mechanisms and control of airborne pathogen transmission. These, amongst a variety of recent studies^17–20^, indicate the flow with and without masks, in coughs, speech and sneezes.

One understudied aspect of droplets formed from human respiratory function relates to droplet formation relates to underlying fluid dynamic instabilities associated with the breakup of liquid streams. These processes directly correlate to large and small droplets. Therefore, transmission relates to the underlying, liquid-gas fluid dynamics during human respiratory function. In general, droplets are formed and dispersed through human respiratory airflow events that generate droplets from saliva/mucus films^9^. These films and larger droplets/fluid ligaments endure flow instabilities that lead to the breakup into smaller droplets when exposed to flow. These breakup processes are strongly tied to relative droplet speed (droplet inertia) and droplet fluid properties. The formation of smaller droplets formation is known to correlate with: (1) lower surface tension, (2) lower viscosity, and (3) increased air-stream speeds. Bringing this into the context of droplets from human respiratory function, the air-stream speed is established from the human respiratory events, i.e., high for a sneeze and low for speech. While the fluid properties of the host’s saliva/mucus drive viscosity and surface tension. The remaining aspect relates to the overall content of saliva and its relation to droplet count. This study aims to understand how these factors relate to pathogen transmission.

Previous studies of saliva generation indicate that both fluid properties and content correlate to human physiology. The saliva secretion rate reduces when humans stressed, depressed, older, or ill^21^. Other factors such as anxiety, fatigue, and headaches also reduce saliva flow rate^22^. Stress can also lead to increased saliva viscosity^23^. These psychological factors relate to those that are symptomatic or prone to illness and, as mentioned above, may lead to reduced transmission rates of pathogens. Lastly, the sex of a human also changes these variables, where women are found to have lesser and thicker saliva^23^. These sex-based differences could be associated with different social behaviors and roles. This leads to an underlying question, are these biological responses a natural mechanism to reduce pathogen transmission?

The overarching hypothesis of this effort is that airborne pathogen transmission is a function of saliva fluid properties and content. We relate this to both human physiology and potential approaches to reduce the transmissibility of airborne pathogens. Specifically, we explore effects of transmissibility from a human sneeze through modifying the saliva in the buccal cavity through: (1) thickening the saliva using colloids and (2) increasing/reducing saliva content using stimulation/anticholinergic. The effort first studies the effect of developing a saliva colloidal suspension (SCS) to alter the droplet formation and breakup mechanisms. The second approach explores altering fluid content. All of these efforts are also compared to a gaiter face mask.

These studies are conducted in the context of induced, real-human sneeze events measured using high-speed flow diagnostics and evaluated for droplet and aerosol content, size, and exposure. The results show clear evidence of a connection between the airborne transmission pathway and buccal cavity saliva properties and content. Specifically, aerosols are reduced with thicker saliva while overall exhaled droplets/aerosols reduced from reduced saliva content.

## Results

The research shows the ability to influence the airborne transmission path through altering saliva/mucus fluid properties using both experimental and numerical methods. Real human experiments are used to provide in-vivo evaluations of droplet character from a strong respiratory event (sneeze) in the context of modern measurement techniques. In order to baseline the studies, the results are compared to a baseline sneeze (no modifications) and a baseline sneeze with a double mask. The mask is based on a neck Gaiter, which was as a baseline mask. In addition, modern numerical methods are used to provide detailed quantification in the context of a controlled sneeze event (i.e., numerically prescribed). Details and results are provided below.

### Effect of saliva colloidal suspension (SCS)

**Figure 1:**
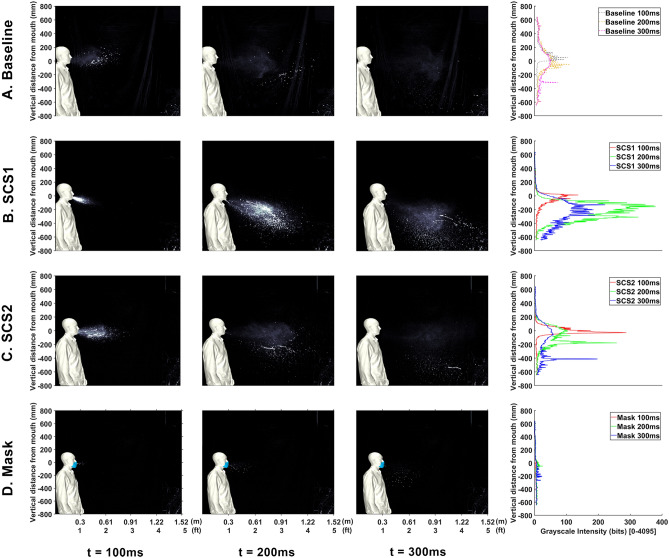
High-speed photography from human sneeze. Cases compare (**A**) baseline, (**B**) SCS1, (**C**) SCS2, and (**D**) masked. Each column represents a different point in time after the event along with the vertical intensity profile indicating overall droplet content.

The first results explore the effect of droplet dispersion from a saliva composed of a colloidal suspension. Such suspensions are known to be less prone to droplet breakup^24^. Also, this may be seen as a viscosity increase, which correlates with films/droplets/ligaments less prone to droplet breakup. High-speed experimental visualizations are presented in Fig.  1, where the baseline sneeze is displayed in Fig.  1A. In the figure, each column presents a progression through time. The last column, presents a vertical image intensity profile that correlates to the overall content of droplets and aerosols as a function of height. This case provides a baseline for comparison of droplets and aerosols expelled from a human sneeze when using various modifiers.

Consider evaluations of the impact of creating a salivary colloidal suspension (SCS) in the buccal cavity. In Fig.  1B,C, an SCS is created using two different types of colloids, C$_{27}$H$_{48}$O$_{20}$ and C$_{36}$H$_{58}$O$_{29}$P$_{2}$, respectively. Table 1 indicates high strain-rate viscosity measurements of the saliva and saliva when the colloids are added (from the same person sneezing). The results indicate a strong ability to alter saliva viscosity. Returning to Fig. 1B,C,the imagery indicates are clear effect that the characters of the droplet content are strongly correlated to saliva fluid properties, and that the SCS leads to both fewer aerosols and larger droplets. The vertical intensity profile (column 4) indicates a downward skew and erratic fluctuations associated with the larger droplets.

**Table 1:** High strain-rate viscosity measurements.

Case	Viscosity	Viscosity increase
Saliva	1.36 cP	–
Cornstarch (SCS1)	1.43 cP	5.1%
Agar agar	2.98 cP	119.1%
Xanthan gum (SCS2)	28.94 cP	2027%
Menthol lozenge	1.79 cP	31.6%
Sugar free menthol lozenge	1.64 cP	20.6%

Next, consider comparisons to conventional mitigation methods of masks. Hence, the first comparison is with a sneeze that is covered with a mask where results are plotted in Fig.  1D. Comparison between Fig.  1A,D show the effectiveness of conventional droplet mitigation approaches. From this image, a clear result is the ability for the mask to reduce the effective distance droplets and aerosols are dispersed. In general, there appears to be a reduction in droplets and an increase in aerosols from the mask. However, these aerosols travel a shorter distance, which enables secondary dispersion mechanisms (such as room currents and/or winds) to more safely dilute the aerosols. Such a result is useful for comparison to present mitigation methods.

**Figure 2:**
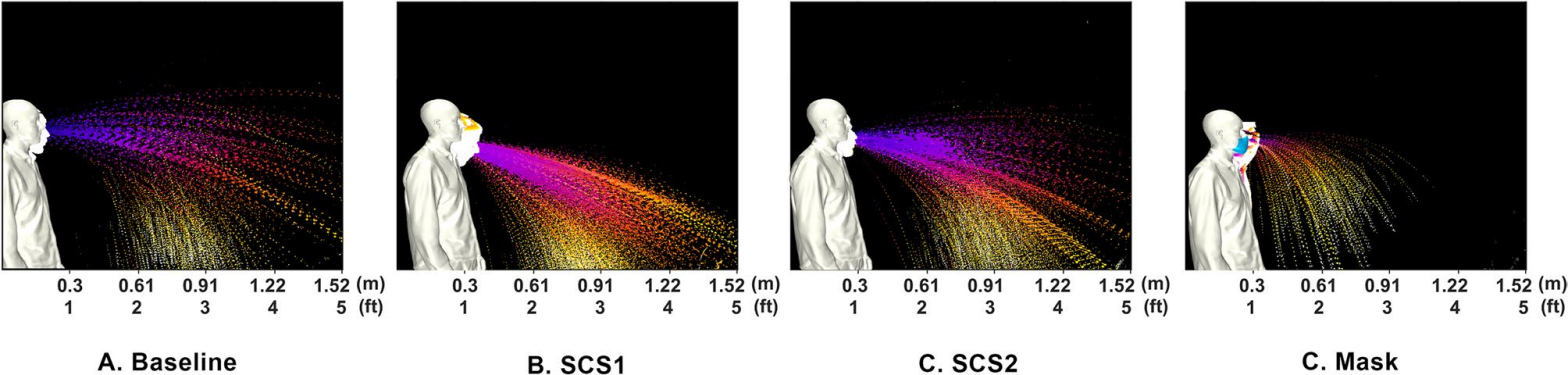
Summary of droplet exposure from Human Sneeze with various modifiers. Cases compare (**a**) baseline saliva, (**b**) SCS1, (**c**) SCS2, and (**d**) mask.

**Figure 3:**
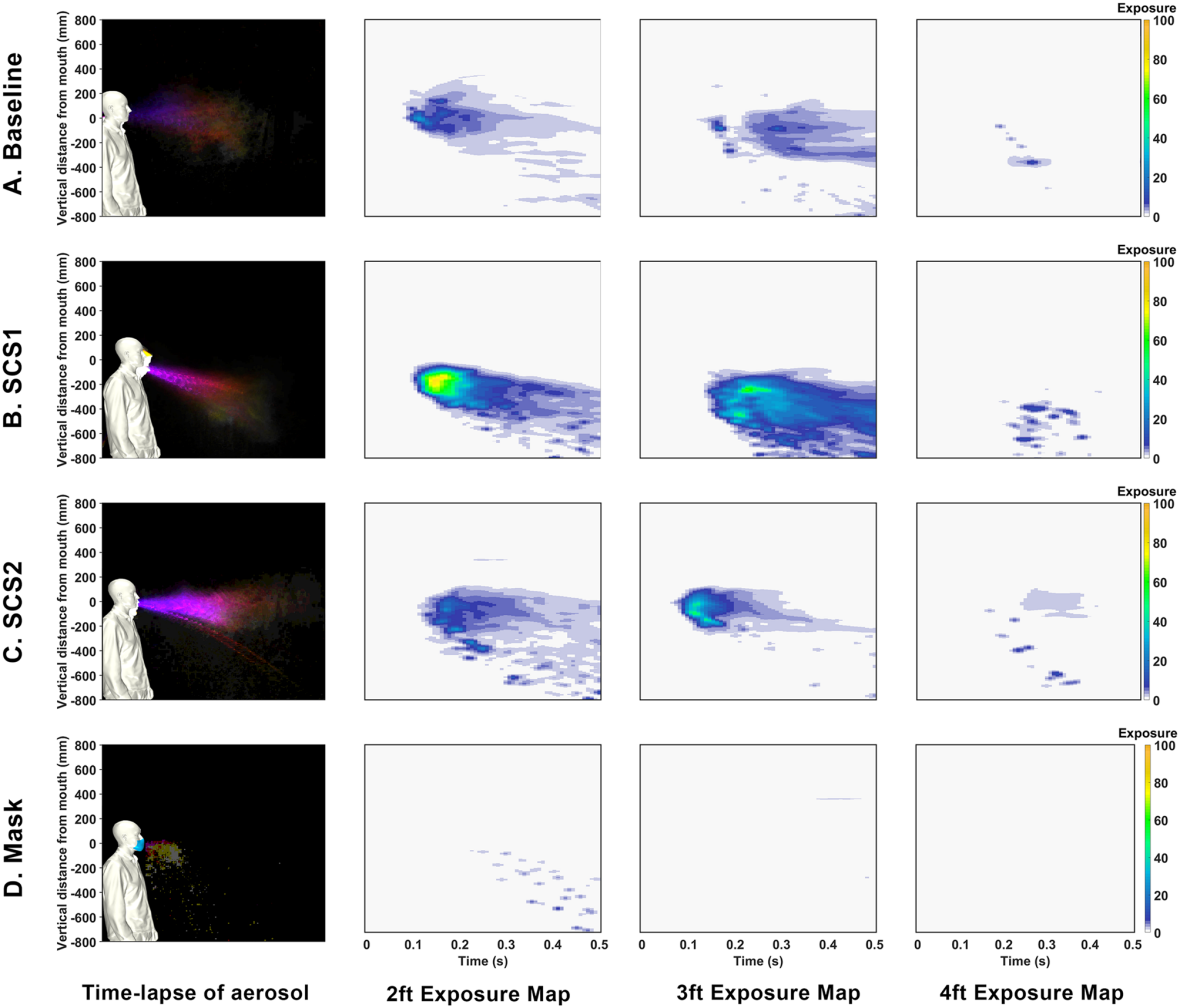
Summary of aerosol exposure from Human Sneeze with various modifiers. Column 1 is a time-lapse colored by time. Column 2–4 indicate exposure levels at 0.61 m (2 ft), 0.914 m (3 ft), and 1.219 (4 ft), respectively. Cases compare (**a**) baseline saliva, (**b**) SCS1, (**c**) SCS2, and (**d**) mask.

In order to improve the understanding, the images are processed such that they separate droplets and aerosols. A time-lapse of the droplets is depicted in Fig. 2 where the time-lapse image represents the overall spatio-temporal droplet exposure in a single image. Here, the droplets are extracted from each image and stacked and colored by time (purple is near 0*s* and white is 0.5 s). These interpretations indicate the overall region that is exposed to droplets throughout the entire duration of the event. These time lapses are performed for droplets in Figs. 2 and 7. Additionally, a similar time lapse of the aerosols are presented in the left subpanel of Figs. 3 and 6.

The spatio-temporal exposure to aerosols is presented in figure Fig. 3. This provides the spatio-temporal exposure to aerosols through the event using a similar time lapse applied to the aerosols (first column) along with a distance dependent exposure map (DDEM) in columns 2–4. Expanding on the evaluation of the DDEM in Fig. 3, these plots qualitatively evaluate aerosols. The plots are based on image intensities from the processed images that focus on aerosols. They are normalized by the peak intensity to provide a qualitative metric referred to as an “Exposure”. These exposures are plotted as a function of vertical space (y-axis) and time (x-axis). These are plotted at various axial distances to provide an assessment of the exposure at distance from a sneeze. These overall assessments are used to qualitatively assess exposure from the human sneeze. In this figure, each sub-figure (row) corresponds to the various test cases, e.g., baseline, SCS 1 and 2, and mask. While each of the columns depict various measure of potential exposure levels to the aerosols. In evaluating the time-lapse of the aerosols (column 1), the results indicate the overall region and intensity of exposure to aerosols. The DDEM helps to add a temporal aspect. DDEMS are created using vertical intensity profiles at a given horizontal distance from the sneeze through time. The result is an aerosol exposure potential (color) as a function of height (vertical axis) versus time (horizontal axis). These DDEMs are generated at 0.610 m (2 ft), 0.914 m (3 ft), and 1.220 m (4 ft), in columns 2, 3, and 4, respectively. Note that the baseline condition is overlaid in grey scale for reference. These figures are used to approximately quantify the bulk exposure of aerosols from a human sneeze.

The results from Fig. 3 indicate that the bulk fluid properties in one’s saliva directly correlate to the character of the droplets and aerosols expelled from a sneeze. In general, in comparing Fig. 3A–C, the content of aerosols appears to be reduced. In evaluating the droplets, in Fig. 2, at increased distances there is a clear rise in the amount of droplets. The SCS droplets appear to have a steeper downward trajectory that can be associated with an increased droplet size. Interestingly, the mask also displays droplets (which was an unexpected, yet repeatable, observation). It is believed that this is a result of coalescence within the mask that forms with the extreme respiratory event. In general, the processed imagery suggests that the SCS leads to more droplets that tend to be larger and appears to deplete the aerosol content. Such an effect is consistent with reducing fluid-dynamic breakup mechanisms.

**Figure 4:**
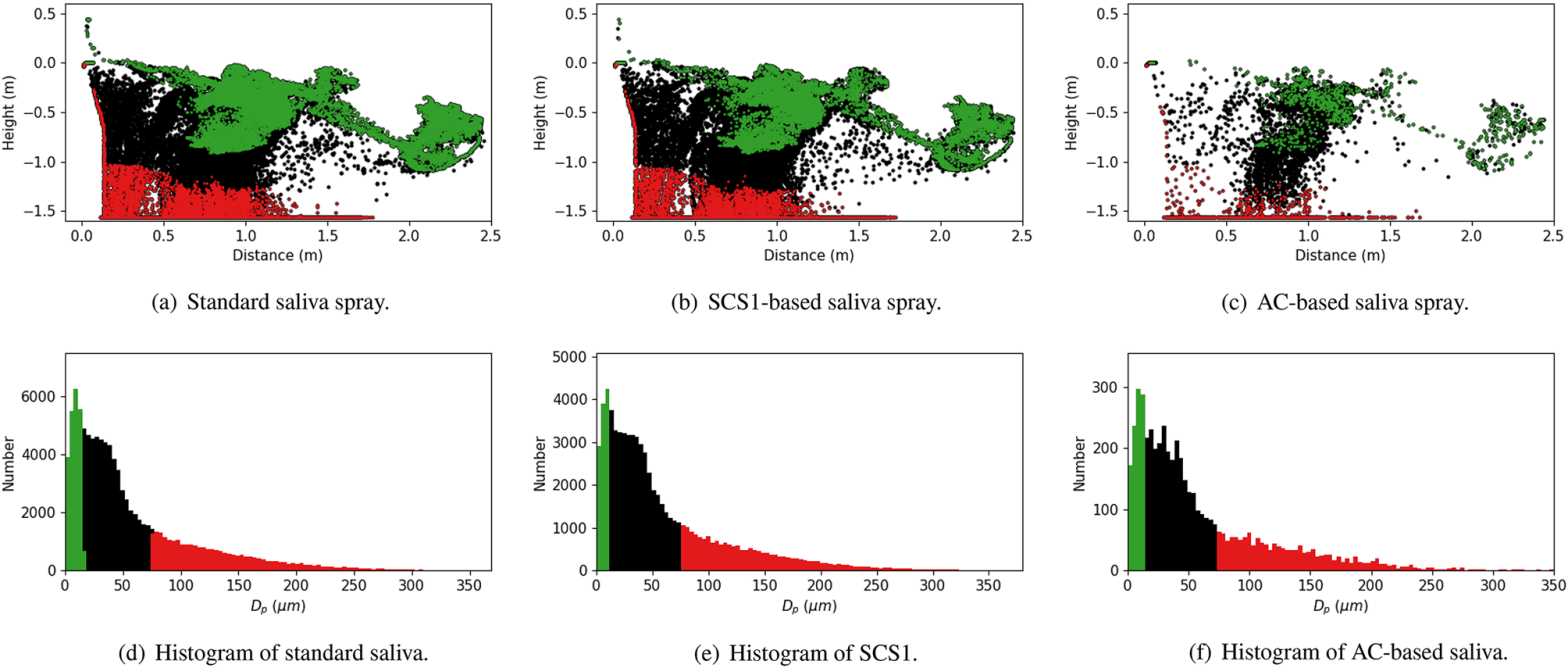
CFD simulations for different liquid droplet properties at 5 s after the sneeze.

In order to better understand this effect, this character is evaluated using computational fluid dynamics (CFD) in the context of Lagrangian droplets in a thermally buoyant sneeze event as described in “Numerical methods” section below. The numerical simulations included evaporation model that accounts for mass transfer between liquid droplets and the surrounding air. Results are provided in Fig. 4, where the properties of the three saliva types are changed in similar directions as the experiments, i.e., from thinning to thickening the saliva. Such a sensitivity study is used to understand the underlying physical mechanisms. Results are plotted in terms of the dispersed droplets 5 s after the sneeze for (a) baseline saliva, (b) SCS1, and (c) AC saliva (discussed in following section). The droplet size distributions, and the corresponding locations, are highlighted by color.

Similar to the experiments, the CFD clearly indicates trends similar to a modification towards larger droplets. Hence, the SCS experiments indicate character similar to having larger droplets that tend to fall from the sneeze plume. Alternatively, a thinner saliva results in smaller droplets that remain suspended with the sneeze plume. Hence, the resulting droplet size distributions shows in a very controlled numerical model to completely alter the saliva content in a plume.

### Effect of saliva content

The second series of studies explores the effect of droplet dispersion from a human sneeze resulting from factors that increase or decrease the saliva content. Essentially, it is hypothesized that the exhaled droplets and aerosols are directly correlated to the amount of saliva in ones mouth. In this approach, we studied these effects by stimulating the salivary glands (SS) using a sugar-based lozenge (or cough drop). To reduce the amount of saliva, an anticholinergic (AC) was explored that is based on Zingiber officinale. In rats, Zingiber officinale does not have a clear effect as it is both documented to increase saliva flow rates^25^, while others suggest an AC effect^26,27^. In our testing, our observation was a “dry mouth” sensation consistent with an AC. Measurements were used to verify this observation where in Unstimulated saliva flow rate measurements provided in Table 2. Hence, Zingiber officinale was used as an AC. The remaining question that remains is how important is the saliva content to droplet content dispersion. This is evaluated using similar approaches and analysis performed for the SCS.

**Table 2:** Unstimulated, saliva flow rate average over 10 min.

Case	Unstimulated saliva flow rate ($\frac{g}{min}$)	Flow rate change (%)
Baseline	0.644	–
Zingiber officinale	0.522	$-21.3$
Gelidium corneum	0.691	10.8
Bacterial polysaccharide	1.160	79.2
Menthol lozenge	1.372	116.7
Sugar free menthol lozenge	0.984	55.6

**Figure 5:**
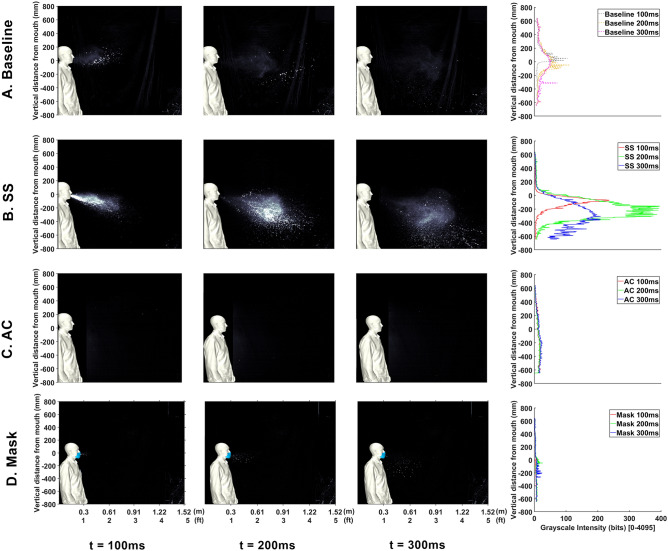
High-speed photography from human sneeze. Cases compare (**a**) baseline, (**b**) SS (sugar-based cough drop), (**c**) AC, and (**d**) mask. Each columns represents a different point in time after the event.

**Figure 6:**
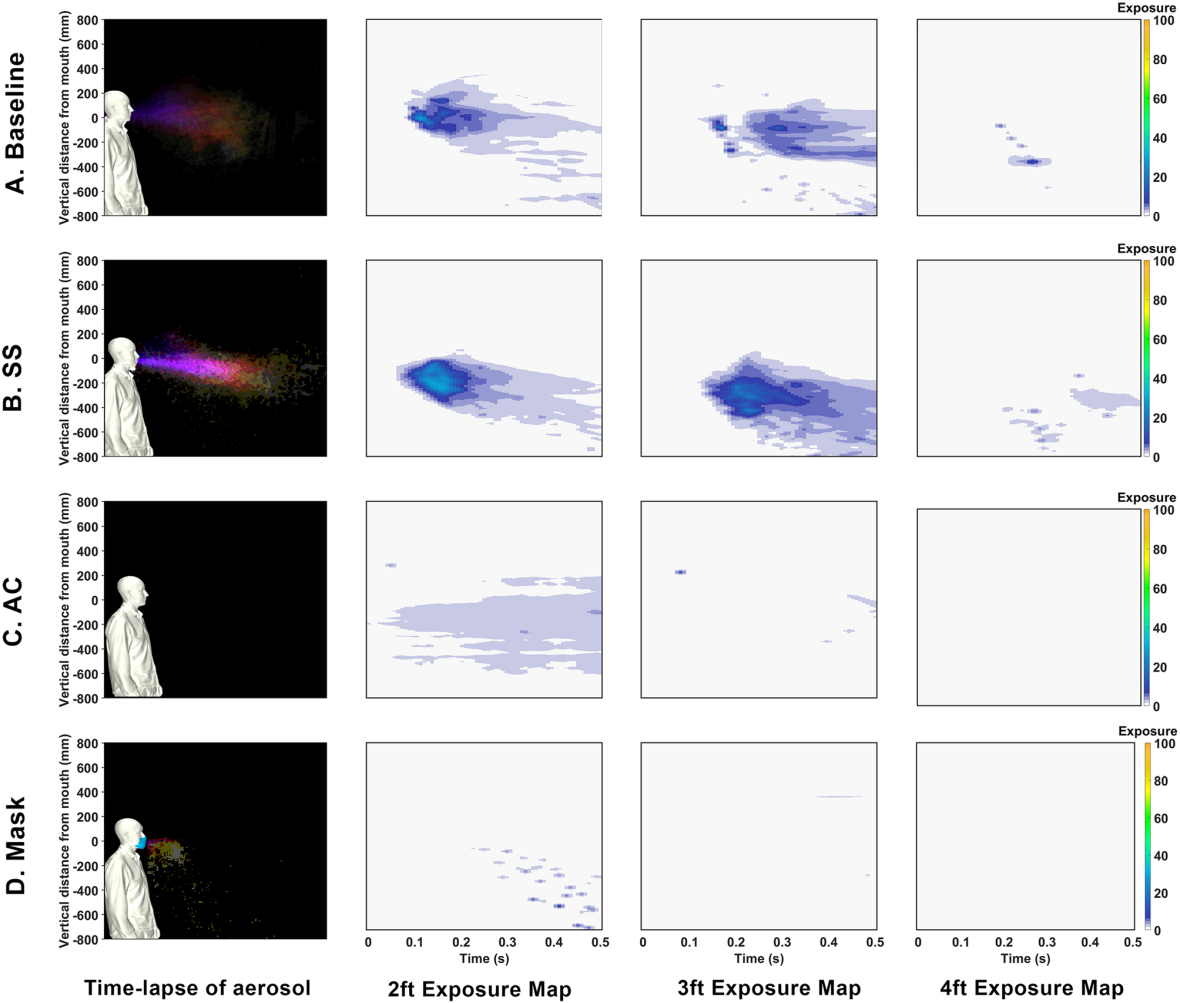
Summary of aerosol exposure from Human Sneeze with various saliva content modifiers. Column 1 is a time-lapse colored by time. Column 2–4 indicate exposure levels at 0.61 m (2 ft), 0.914 m (3 ft), and 1.219 (4 ft), respectively. Cases compare (**a**) baseline saliva, (**b**) SS, (**c**) AC, and (**d**) mask.

**Figure 7:**
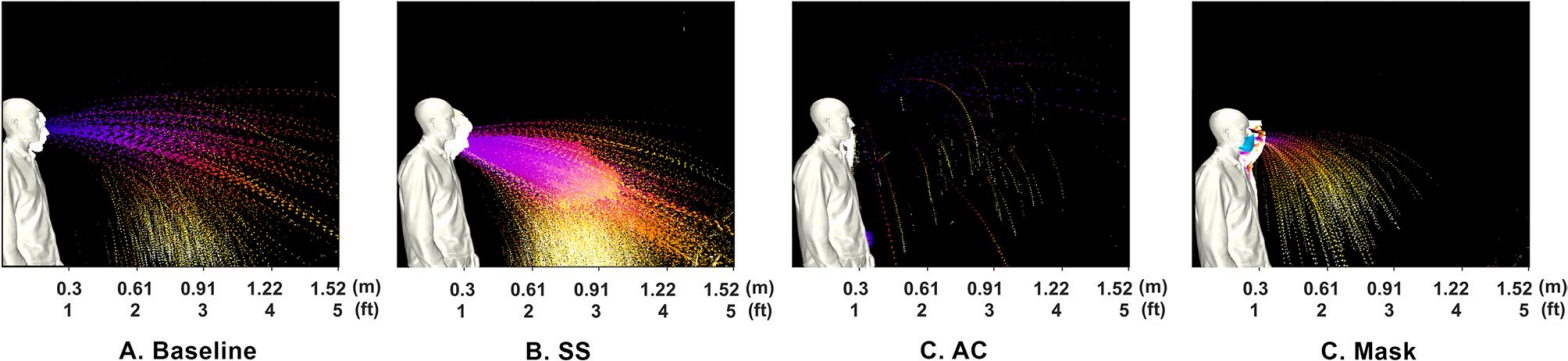
Summary of droplet exposure from Human Sneeze with various saliva-content modifiers. Cases compare (**a**) baseline saliva, (**b**) SS, (**c**) AC, and (**d**) mask.

In Figs. 5, 6 and 7 are the imagery, processed aerosol content, and processed droplet content. These figures compare the baseline and a baseline, SS, AC, and mask. In Fig. 5, the effect of overall saliva content in ones mouth is clearly highlighted in the imagery. In Fig. 5C, a dramatic reduction in the formation of droplets and aerosols is observed from the AC. In contrast to a conventional SS, there is a dramatic sensitivity to the droplets/aerosols expelled as they relate to the content of saliva in the mouth. This finding suggest a strong relationship between the overall liquid content in the buccal cavity in relation to the dispersion of droplets.

The processed images, in Fig. 6, begin to quantify the effects. In this figure, the aerosols are plotted in the first column and the DDEMs in columns 2–4 at various distances. For the AC case, these plots clearly indicate a complete reduction in aerosol exposure throughout the event. In terms of evaluating droplets, in Fig. 2, a similar time-lapse image is used for each case. In Fig. 4c, are the CFD simulations to represent the AC case. The CFD results highlight the general result that the emission is reduced, however, the distance traveled is not strongly affected. These figures are used to approximately quantify the bulk exposure of saliva from a human sneeze.

### Dispersion and exposure quantification

**Figure 8:**
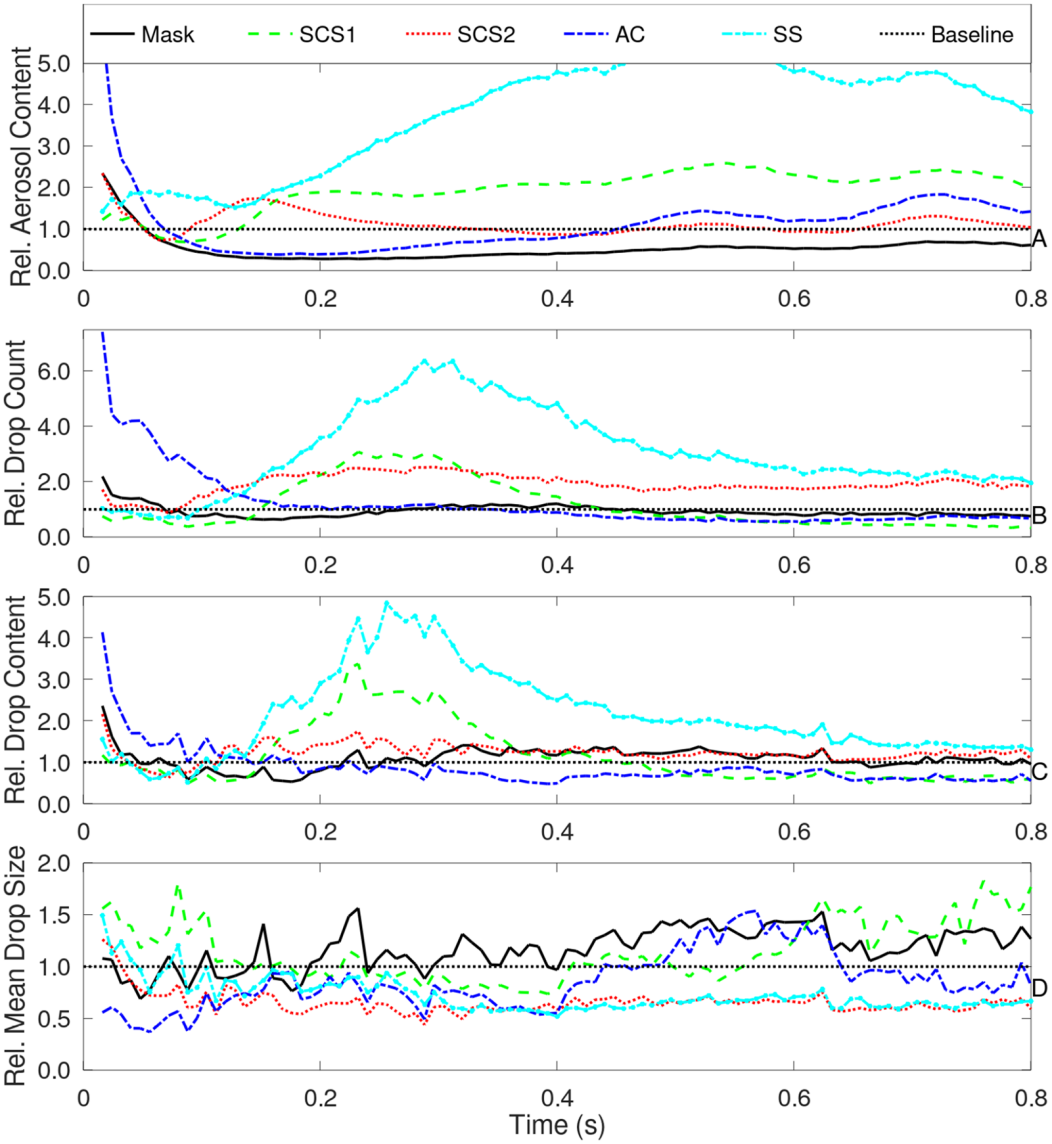
Image processed estimation of overall measures of aerosols and droplets with various modifiers including a baseline, mask, SCS1, SCS2, SS, and AC. The overall aerosol content (upper), droplet count or number of droplets (2nd row), droplet content or area exposed to droplets (3rd row), and mean droplet size (4th row) are all plotted versus time. Results are normalized by the baseline.

Within the 1.52 m $\times$ 1.52 m (5 ft $\times$ 5 ft) imaging region, the results are processed in ImageJ^28,29^ and MATLAB^30^. The image processing aims to identify droplets, aerosols, and quantify (relative to the baseline) the content of each condition. The overall procedures of the normalization are discussed in the methods. Normalization using the baseline human sneeze is performed for two reasons. First, relative effects are most critical as it both is more consistent (i.e., reduces human-to-human or random variations) and still relates to actionable efforts. It also eliminates issues associated with indirect measurements. For example, estimating droplet size is difficult from light distortion. However, relative droplet size is more easily measurable as the uncertainties (from light distortion) are also normalized and generally reduced. Hence, the total content in the image, in terms of levels relative to the baseline as a function of time, are plotted in Fig.  8. In Fig.  8 is a summary of the aerosol and droplet content. In this figure, it can be observed that the mask reduces the overall aerosol content, while having roughly the same number of droplets. Such findings are consistent with previous studies of a neck gaiter mask^31^. Comparatively, the SCS1 case increases aerosol and droplets. The more viscous, SCS2 case, decreases total aerosols while increases the number of droplets. These more abundant droplets are, on average, smaller but also have much larger droplets. The SS case (cough drop), indicates a near 5-fold increase in droplet and aerosol content. Lastly, the AC observes a drop in droplet and aerosols. Hence, the results appear to indicate potential for fluidic control of aerosol dispersion associated with both content and viscosity.

**Figure 9:**
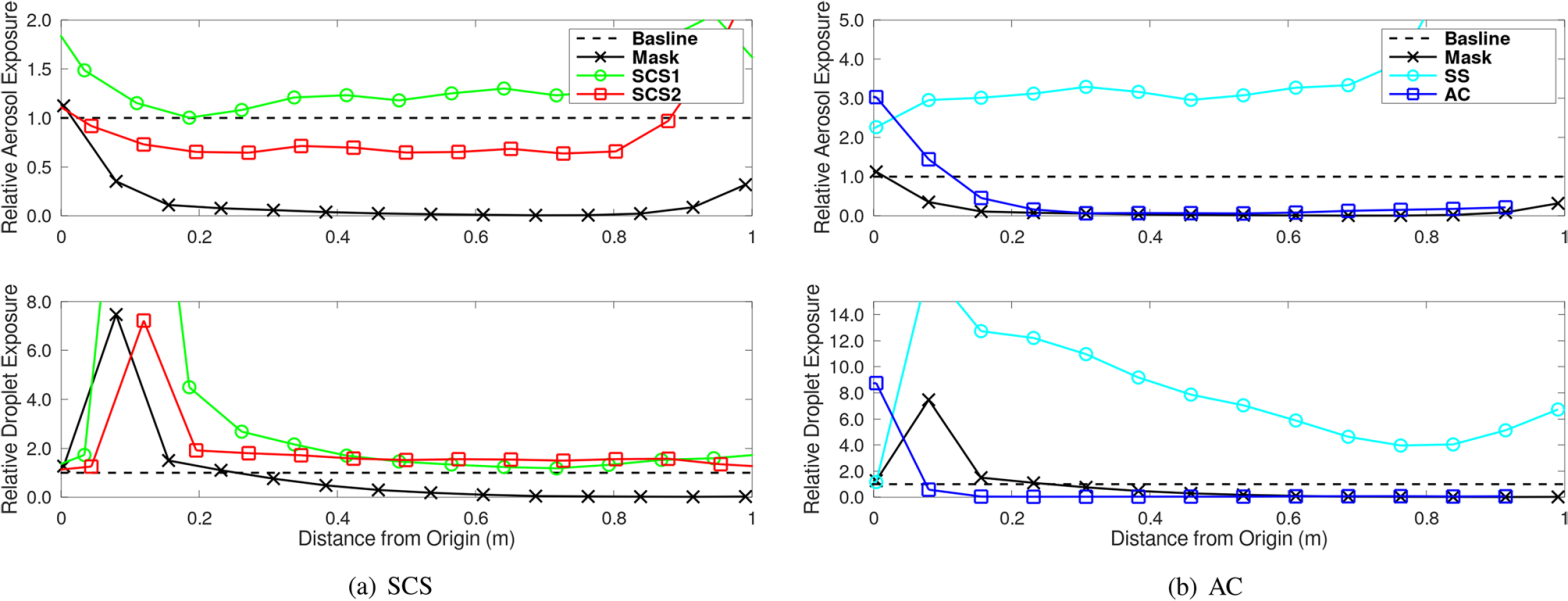
Relative exposure to aerosols (upper subplot) and droplets (lower subplot) as a function of distance using (**a**) SCS and (**b**) AC.

In order to quantify the overall exposure potential as a function of distance from the origin (the human mouth), the DDEMS for aerosols and droplets are integrated over time to estimate exposure levels. Similar to before, the DDEM values are normalized by the baseline to provide a relative exposure level of the aerosols and droplets. Results are provided in Fig. 9. Note that these figures are adjusted to correlate in space such that the mouth is at the origin (0 m). The effect of colliods are presented in Fig. 9a, and compared to the baseline and mask. Despite the neck gaiter not impacting the number of droplets, there is an initial rise followed by a subsequent drop in droplet exposure. Similarly, the aerosol exposure also decreases with distance. This indicates the neck gaiter, despite increasing droplets^31^ and aerosols, effectively reduces exposure. The colloids appear to consistently increase droplet exposure, while indicating an ability to reduce aerosols. Fig. 9b are the effects of the SS and AC. The SS indicates a remarkable increase in both the droplet (4–12 times) and aerosol exposure (3 times). Alternatively, the AC indicates the ability to mitigate these rises.

**Figure 10:**
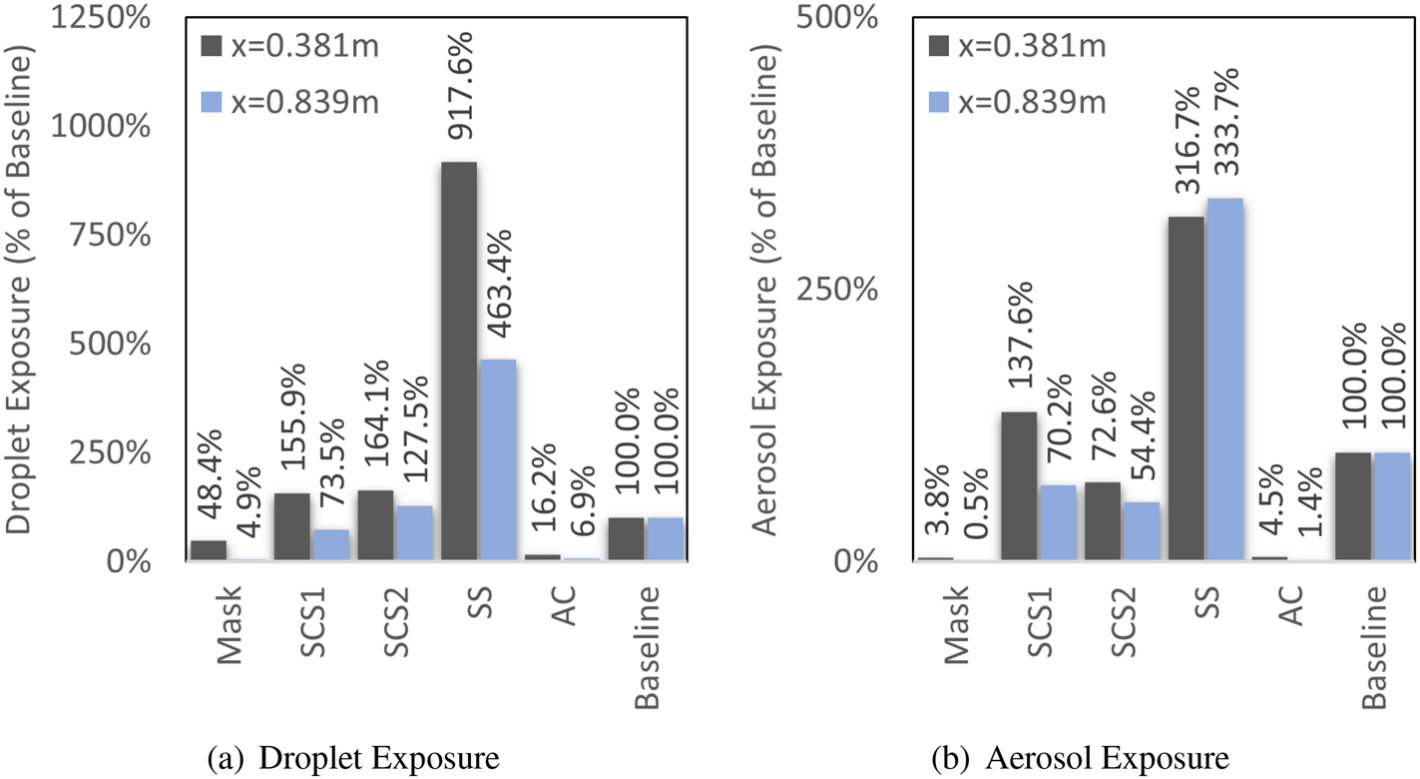
Overall potential of exposure to aerosols (**a**) and droplets (**b**) as a function of distance and normalized by a baseline.

To summarize the results, bargraphs comparing exposure at two distances (0.381 m and 0.839 m) are provided in Fig. 10. The neck gaiter mask indicates to be very effective with a $48.4\%$ (0.381 m) and $4.9\%$ (0.839 m) of the droplets of the baseline. Additionally, the aerosols are $3.8\%$ (0.381 m) and $0.5\%$ (0.839 m) compared to the baseline. The SCS cases indicate a sustained rise in droplet exposure (up to $155.9\%$ of baseline), however, there appears to be a drop in aerosol levels (up to $54.4\%$ of baseline). The SS indicates a a substaintial rise in droplets (over $463.4\%$ of the baseline) wiht a corresponding greater than $316\%$ rise in aerosols. Lastly, the AC appears to perform similar to the mask. Results indicate a substantial decrease in droplets, i.e., $3.8\%$ (at 0.381 m) and 0.5% (at 0.839 m). While aerosols are 4.5% (at 0.381 m) and 1.4% (at 0.839 m). In summary, while masks are effective, it is also important to consider fluidic aspects that can drive or prevent aerosols and droplets.

## Discussion

Pathogen transmission occurs through a variety of mechanisms. In this work, droplet formation was studied to explore if the amount can be reduced as well as altered so that aerosols are reduced and they droplets fall to the ground. Hence, large droplets are associated with fomite transmission paths that can potentially infect the gastrointestinal tract. Whereas aerosols can be easily inhaled and can infect the respiratory tract. Influenza is generally assumed to infect the respiratory tract, while SARS-CoV-2 is primarily suspected to infect the respiratory tract, but can also impact the gastrointestinal tract^32^. The overall effort indicates that these transmission paths are potentially altered through factors relating to a host salivia properties and can also be modified using formulated compounds to reduce transmission rates.

The overall findings suggest that saliva that is higher in content and less viscous directly correlate with transmissibility of droplets (and likely airborne pathogens). Reducing the content of saliva appears to reduce both aerosol and droplet content, which respectively correlate to airborne and fomite transmission. Saliva viscosity appears to reduce aerosols and create more droplets, which reduces airborne transmission, while potentially increasing fomite transmission. Based on a series of studies on saliva flow rate and rheology, a human will naturally respond to illness and stress such that they naturally prevent transmissibility. These findings are inconsistent with epidemiological models that typically model symptomatic hosts with increased transmission rates. Recall that symptomatic hosts cough and sneeze more. Such events create many more droplets and aerosols than speech. Hence, it is proposed that human physiology actively responds through reducing transmissibility per an event in symptomatic hosts. Additionally, factors such as sex may also play a role in transmission. As previously mentioned, women having a naturally lower saliva content that is thicker may also have a lesser tendency to transmit pathogens^23^. In general, the present study finds that women, depressed/stress, and ill individuals have a natural tendency to reduce the transmission rate airborne pathogens.

Lastly, it was also found that transmissibility can potentially be enhanced or controlled through saliva stimulation. In this effort, food-grade compounds were utilize to reduce transmissibility to levels similar to a mask (in the context of a human sneeze). It was also observed that stimulating saliva (using a cough drop) significantly increases transmission potential. It is possible that such processes can be extended to provide alternative paths to reduce transmission rates of pathogens. One example may be using these compounds in collaboration with a mask which, if transmission rates are significantly reduced, could lead to safely relaxing social distancing. In addition, the usage of sugar-based cough drop needs further attention as it generally is suggested (by the CDC and others) for reducing cough symptoms. These cough drops may reduce the number of coughs, but also increase the droplets/aerosols formed in a cough. Hence, can act to both decrease and increase the transmission of a pathogen. In general, it is proposed that considering the character of the saliva be considered as another tool to combat the COVID-19 pandemic and other pathogen-driven disease.

## Methods

### Experimental methods

The study was approved by the University of Central Florida Institutional Review Board (IRB), Orlando, Florida, United States, and meets the requirements established in 45 CFR 690.118. All methods were performed in accordance with these guidelines and regulations. All participants (over 18 years old) were provided informed consent of the experiments and releasing data to open-access publications.

The experiments are based on high-speed flow diagnostics to quantify droplets and aerosols. The setup of the experiment involves a high-speed cameras and six light sources that are used to acquire measurements of a subject sneezing^14,33^. The images are captured at 5400 frames per second, with a total recording time of 1 s, and have a spatial resolution of 1.2   mm/pixel for a total span and height of 1230 $\times$ 1230 mm ($5 \times 5$ ft). Each light source consists of a 750-W halogen bulb emitting light from 400 to 1800 nm with a peak at 750 nm(17). Three light sources are spaced equally along the horizontal distance above the region of interest pointed downward and the other three are below pointed upward. This allows for sufficient light scatter of the droplets exiting the subject’s nose and mouth with even illumination across the recording region of interest ($\pm 7$ of an intensity count). The light scatter allows for tracking of droplets through time. Removing geometric bias is also performed to account for the inability to control a position during a real human sneeze. The subject’s spatial location is marked with respect to the recording equipment to ensure consistency in location across all test cases. The location of the subject’s head can deviate by 100 mm (4 in.) horizontally and vertically at the start of the event and can move up to 205 mm (8 in.) during the event. The maximum deviation angle of the subject’s head at the start of the event is 25$^{\circ }$ between cases and can deviate as much as 6$^{\circ }$ during the event. These discrepancies are accounted for by correcting each image back to an origin and orientation, so that each test case can be directly compared.

### Image processing

The high-speed, high-resolution imagery is processed within ImageJ^28,29^ and in-house MATLAB processing^30^. ImageJ is a well recognized image processing tool that consists of a library of various algorithms that are used to: (1) estimate aerosols (concentration and intensity), (2) estimate droplets (size and count), (3) estimate exposure to saliva. The images are processed as follows. Step 1: A region of interest is selected by isolating regions associated with the droplets and aerosols exhausted from a sneeze. Step 2: Background subtraction is performed to mitigate spurious sensor noise and to heighten the gradient between the background and droplet intensities. Step 3: Intensities through time are tracked at specific spatial locations of 2, 3, and 4 ft to generate an exposure map associated with a human sneeze. The individual exposure maps in Figs. 3 and 6 are generated by normalizing the spatial aerosol content for each saliva modifier by their respective average intensity. The colorbar shown on the right of the images represents the resulting normalized exposure from 0 to 100 units. Note that the colorbar applies to the exposure maps only, not the time-lapse images on the left of Figs. 3 and 6. Step 4: The stack is then filtered (FTT-based, band-pass filter over scales larger than 20 pixels) to isolate the droplets. This resulting, filtered, image is then binarized using the Lin method to isolate the droplets. The droplets are then counted and evaluated using ImageJ’s particle analyzer. Step 5: Aerosols are estimated by subtracting the droplets from the overall stack of images. These aerosol images are then evaluated/measured within imageJ.

The image processing provides output that are qualitative in nature which is a result of various underlying factors. To name a few, the output is two-dimensional evaluations of a three-dimensional line-of-sight measure, this, and other factors, are embedded in the processing. In order to expand these data to quantitative results, we take an approach that normalizes each results by the baseline sneeze (i.e., no additives). These relative measures include the relative droplet count, $N_{DROP}^*$, which is evaluated as the droplet count normalized by an equivilent droplet count in the baseline, or

1 $$\begin{aligned} N_{DROP}^{*}=\frac{N_{DROP}}{N_{DROP,0}}. \end{aligned}$$

Here, the image processing counts the number of droplets in the case of interest, $N_{DROP}$, and normalizes the number by those from the baseline case, $N_{DROP,0}$. Note that the subscript 0 implies operation on the baseline data. Similarly, the mean relative droplet size, ${\overline{D}}^*$, can be computed as the pixel-based droplet length scale, normalized by the baseline, pixel-based droplet length scale, or

2 $$\begin{aligned} {\overline{D}}^{*}=\frac{N_{DROP,0}}{N_{DROP}}\frac{\sum _i^{N_{DROP}}{ \sqrt{ N_{pix,i} } }}{ \sum _i^{N_{DROP,0}}{ \sqrt{ N_{pix,i,0} } }}. \end{aligned}$$

Here, the parameter $N_{pix,i}$ is the pixel count for droplet *i*. The overall droplet content is also evaluated in a relative sense, indicated by $A^*$, which is computed as

3 $$\begin{aligned} A^{*}=\frac{\sum _i^{N_{DROP}}{N_{pix,i}}}{\sum _i^{N_{DROP,0}}{N_{pix,0}}}. \end{aligned}$$

Lastly, the overall aerosol content can be provided by integrating the image intensity of the case of interest, by the same integration of the baseline. The normalized aerosol content, $AC^{*}$, is given as

4 $$\begin{aligned} AC^{*}=\frac{N_{pix,0}}{N_{pix}}\frac{\sum _i^{N_{pix}}{I_i}}{\sum _i^{N_{pix,0}}{I_{i,0}}}. \end{aligned}$$

Here $I_i$ is the individual pixel intensity. These image processing methods enable quantitative assessment of effects form various factors.

### Numerical methods

The whole description of the physical, mathematical, and numerical models used to obtain the CFD results in the present work is described in recent study focusing on understanding the fluid dynamics and human physiology factors driving droplets dispersion from a human sneeze^34^. The present effort expands on that study using an Euler–Lagrange approach to treat the continuous air phase interacting with the dispersed liquid droplets that formed the spray generated from the sneeze event. This type of approach has been widely used to model spray formation in many configurations^35–40^. Given the similarities associated with physical conditions and air-droplet interactions, Euler–Lagrange approach is suitable to investigate the spray formation generated from sneeze events.

In order to provide a realistic conditions possible, we approximate the sneeze in the context of a human body within a room. The human body model was used to evaluate how it affects the dispersion of droplets during a sneeze. The human model, with a height of 1.78  m, is located at the center of a cylindrical domain with a radius of 3.0  m and height of 3.0  m. The sneeze is driven by an inlet boundary condition at the bottom surface of the throat of the human model. An artificial nasal passage is also included to improve the realism of the model. Despite a number of approximations, the overall model has a reasonable resemblance to a real-human sneeze. We did not consider droplet dynamics associated with droplet-droplet interactions (coalescence/stripping). The assumptions that droplets do not interact with each other is valid in the dilute concentration regions with a low probability of collision^41,42^ exterior to the mouth. However, it may present deficiencies inside the upper respiratory tract and close to the mouth/nasal passages, which are neglected in the present work. We included mass transfer between droplet and the surrounding air through evaporation/condensation model. Regarding mass transfer due to evaporation, we assumed that the relative humidity is $50 \%$ (typical value for an air conditioned environment) and the air coming out from the mouth has a relative humidity of $80 \%$, which is typically found in human exhaling^43^. We used the multi-component droplet evaporation model to transfer liquid mass from droplets to the air and vice-versa. This models considers that the driving force for evaporation and condensation is the departure from equilibrium of the liquid-vapor system^44^. However, due to the ambient conditions, temperature $T_{amb} = 23\,^\circ$C and relative humidity, the droplets presented small changes in size. This results agree with a recent investigation about the weather impact on airborne coronavirus survival^45^, in which the authors found that the spray distance and droplet mass loss is less affected in ambient with low temperature (considering a temperature range of 0–40 $\,^{\circ }$C) and high relative humidity, which corroborates our assumption.

Based on previous experimental work^46,47^, we assumed the total volume of injected air is 1.2   l. The air is injected from the bottom surface of the throat according to a temporal velocity profile, adapted from work of Gupta^48^, considering the time of sneeze is 500   ms^47^. For the simulation, three liquid droplets were considered, differentiate to each other by the following properties density, $\rho$, viscosity, $\mu$, and surface tension coefficient, $\sigma$: saliva with $\rho = 997.56$, $\mu = 8.9 \times 10^{-4}$ Pa s, and $\sigma = 0.072$  N/m; thickened saliva with $\rho = 1197.07$, $\mu = 1.16 \times 10^{-3}$   Pa s, and $\sigma = 0.108$  N/m; and thinned saliva with with $\rho = 798.05$, $\mu = 6.2 \times 10^{-4}$ Pa s, and $\sigma = 0.036$ N/m. The droplets were injected from the bottom surface of the buccal cavity (tongue). The total amount of droplets injected was 2 ml, which correspond to $0.0167\%$ of the gas flow rate.

The representative equations of mass, Eq. (5), momentum, Eq. (6), and energy, Eq. (7) for the gas phase are discretized using the finite volume method^49^, with temporal and spatial interpolations of second order. In the case evaluated here, we have wall-attached flow on the buccal cavities that may be turbulent, along with free-turbulence from the sneeze plume. With this variation of scales, we choose to use the hybrid Reynolds-Averaged Navier Stokes/Large Eddy Simulation approach referred to as Detached Eddy Simulation^50^. Such approach is conventional to use in such scenarios, however, in the context of sneeze dynamics turbulence model choice is not yet to be well established.

5 $$\begin{aligned}&\frac{\partial \rho }{\partial t} + \frac{\partial \rho {\hat{u}}_i}{\partial x_i} = 0, \end{aligned}$$

6 $$\begin{aligned}&\frac{\partial \rho {\hat{u}}_i}{\partial t} + \frac{\partial \left( \rho {\hat{u}}_i {\hat{u}}_j \right) }{\partial x_j} = -\frac{\partial {\hat{p}} }{\partial x_i} + \rho \mathbf {g} +\frac{\partial (\mu {\hat{S}}_{ij} + Tm_{{ij}})}{\partial x_j}, \end{aligned}$$

7 $$\begin{aligned}&\frac{\partial \rho c_p {\hat{T}}}{\partial t} + \frac{\partial \left( \rho c_p {\hat{u}}_i {\hat{T}} \right) }{\partial x_j} = k\frac{\partial ^2 T}{\partial x_j}. \end{aligned}$$

In these equations, $\rho$ is the density, which is a function of the temperature according to the ideal gas equation; ${\hat{u}}_i$ is a filtered velocity field; ${\hat{p}}$ is the pressure field; ${\hat{S}}_{ij} = \left( \frac{\partial u_i}{\partial x_j} + \frac{\partial u_j}{\partial x_i}\right)$ is the shear stress tensor; $Tm_{ij} = f_\Delta \left( \frac{\Delta }{l_k} \right) 2 \mu _t {\hat{S}}_{ij}$ is the Reynolds stress tensor, which is function of a damping function from the DES model, the local measure of the grid size, $\Delta$, the turbulent length-scale, $l_k$, the turbulence viscosity, $\mu _t$, and the shear stress tensor;${\hat{T}}$ is the filtered temperature; and $c_p$ and *k* are, respectively, the specific heat and thermal conductivity coefficients of the air.

The droplets are solved via a Lagrangian approach. The model is driven by Newton’s second law to calculate the droplet acceleration coupled to aerodynamic drag ($F_{d_{i}}$), lift ($F_{l_{i}}$), buoyancy ($F_{{w}_{i}}$), and pressure gradient forces ($F_{p_i}$). Droplet velocity and position are obtained from the solution to the ordinary differential equation, Eqs. (8) and 9,

8 $$\begin{aligned}&m_d \dfrac{du_{d_i}}{dt}=F_{d_{i}}+F_{l_{i}}+F_{{w}_{i}} + F_{p_i}, \end{aligned}$$

9 $$\begin{aligned}&\dfrac{dx_{d_i}}{dt}=u_{d_{i}}, \end{aligned}$$

where the subscript *d* refers to droplet; *u* and *x* are, respectively, the droplet velocity and position; *m* is the droplet mass; and subscript *i* indicates the three components of a vector. Other forces such as virtual mass and Basset forces, are normally not relevant because of the high liquid/air density ratio. In terms of air and droplets interaction, the droplets are transported by the air flow, but they do not affect the gas flow, which is known as one-way coupling. The droplet breakup is accounted via Taylor Analogy Breakup (TAB) method^51^.
